# Transition into distance education: A scoping review protocol

**DOI:** 10.1371/journal.pone.0298882

**Published:** 2024-02-23

**Authors:** Roxana Schweighart, Michael Hast, Caroline Trautwein

**Affiliations:** IU International University of Applied Sciences, Erfurt, Germany; University of Johannesburg, SOUTH AFRICA

## Abstract

The number of students choosing to complete their studies online rather than in-person is on the rise. The transition to university is crucial for subsequent success, regardless of whether the learning is done face-to-face or remotely. If students can effectively handle the critical requirements associated with the transition to distance learning, their likelihood of success increases. However, thus far, little information is available on this topic. To gather existing sources, we will conduct a scoping review according to the guidelines of the research organization JBI. The review aims to systematically record, evaluate, and subsequently present the existing body of literature regarding the subject "transition into distance education". By answering the research question, "What is known about transition into distance education in higher education?" we hope to shed light on individual experiences, challenges, adaptation strategies, life situations, etc. of distance learners in a higher education context. The review will identify and categorize relevant concepts and terminologies in the research field, as well as outline the content-related boundaries. The findings derived from the scoping review will provide conceptual clarification, summarize the given theoretical and empirical knowledge in the field and offer practical implications for designing the initial phase in distance education. This article presents the a-priori study protocol that provides a detailed outline of the planned methodology for conducting the Scoping Review.

## Introduction

Digital learning and distance education programs are becoming increasingly prevalent. In 2017, there were 10.6 million individuals worldwide participating in distance education. This number has drastically increased to 27.4 million by 2023. Parallel to the surge in distance learners, revenues tripled within those six years [1]. But what exactly does the term "distance education" entail? A widely accepted definition has been provided by Simonson et al. [2]: "Distance Education is institution-based, formal education where the learning group is separated, and where interactive telecommunications systems are used to connect learners, resources, and instructors". Distance education began in the 18^th^ century. During this time, lecturers and students communicated through the postal service. The evolution of distance education in the 19^th^ and 20^th^ centuries expanded through radio, television, and telephone. In the second half of the 20^th^ century, satellite television, personal computers, and the advent of the internet further improved distance education. In the 21^st^ century, the rapidly increasing use of the internet contributed to today’s advanced online learning modules [3]. Particularly during the Covid-19 pandemic, the significance of distance education in all educational settings—from elementary school to university—has surged tremendously. Globally, the majority of higher education institutes transitioned to distance learning with the aim of reducing the risk of infection for students and staff [4].

Studies by Brown et al. [5] and Mittelmeier [6] illustrate that the living conditions, initial experiences, and adaptation strategies of students entering the distance learning environment for the first time are not merely black and white, but rather, extremely complex. On the one hand, this form of studying is characterized by advantages for the learners: students appreciate the high flexibility, the autonomy, the variety of teaching materials, the elimination of travel costs and routes, the possibility to combine study and work, and the pleasant home environment while learning [7, 8]. On the other hand, students also identify drawbacks to distance learning: issues with concentration and motivation, challenges with navigating the digital world, lack of face-to-face communication with peers and lecturers, difficulties in executing practical tasks, and disruptions—both from other individuals and of a technical nature [5, 7].

Moore [9], in his ‘theory of transactional distance’, defines distance education not simply as a geographical separation of learners and educators, but as a pedagogical concept with a varying degree of transactional distance as a potential disadvantage. Here, the term "transactional distance" refers to the psychological or communicative gap that exists between learners and lecturers, specifically in the context of distance education—a gap that could impede successful digital learning [10]. Moore [9] identifies three underlying variables—dialogue, structure, and learner autonomy—that interact within a system to determine the extent of transactional distance. This distance is particularly minimal in less structured learning environments that facilitate a high degree of dialogue, such as synchronous online lectures or online workshops. Conversely, a high degree of distance occurs in highly structured settings—when individual learner needs cannot be met, and interaction is sparse. This normally occurs in asynchronous learning scenarios, such as when texts or videos are provided to students. Furthermore, the greater the transactional distance between the learners and lecturers, the more autonomy and self-management is required from the learners. Moore’s [9] theory can help distance education lecturers in designing their courses by considering his insights to curtail transactional distance and augment learning outcomes.

When we consider empirical research on academic success in distance learning, it clearly presents mixed results: On one hand, some studies show that distance learners do not underperform, for example, in terms of grades, compared to students in traditional learning settings [11, 12]. On the other hand, there is evidence suggesting that distance learners are more likely to fail, or dropout compared to face-to-face students [13, 14].

Regardless, both in face-to-face and digital learning environments, the study-entry phase is critically important for successful learning [15, 16]. Gale and Parker [17] refer to this entry as a transition phase that requires students to develop a "capability to navigate change". The transition into higher education is drawing increasing attention globally [15]. Related research describes transition as a turning point in an individual’s life course [18], or as an ongoing and progressive shift [19]. This phase is crucial: if students can successfully manage its critical requirements and develop significant study skills at the start of their studies, then successful studying becomes feasible [20]. Many students find the beginning of their studies challenging [20], which is evidenced by elevated dropout rates early on in their educational journey [21]. The critical requirements that students face in their first year can be classified into four dimensions: (1) personal requirements, such as dealing with financial issues or coping with workload; (2) organizational requirements, like creating a schedule or the need to orient oneself; (3) content requirements, for instance, acquiring academic language skills; and (4) social requirements, such as establishing new social relationships or interacting with academic staff [20]. In a study conducted at the Polytechnic University in Bucharest, 30 percent of the surveyed 18- to 21-year-olds considered their adjustment to the context of higher education to be unsuccessful due to crucial challenges, mainly organizational ones [22]. However, the factors regarded as critical differ among students [23], reflecting the growing heterogeneity of the student population [24, 25]. Traditional undergraduate students, who generally enroll in a residential higher education program immediately after high school, vary in their socio-demographic characteristics, as well as in their study expectations, their methods of adapting to the new experiential space of higher education [23], and in other personal variables such as self-efficacy expectations [26].

Non-traditional students, conversely, differ from traditional students in terms of age, living situation, and study expectations [27]. While there is no commonly accepted definition of non-traditional students, Wolter [28] identifies five different, but by no means distinct, interpretations of the concept: (1) individuals with unconventional or non-linear biographies; (2) those who obtained their university entrance qualifications through special access routes and admission procedures; (3) first-year students who are outside of the typical age range for starting university; (4) individuals in flexible study programs; and (5) individuals from groups that are underrepresented in the higher education system. For these individuals, non-governmental, private universities and their offered distance learning programs are particularly relevant, as they often enable access to higher education [29].

A recent dissertation provides evidence that cultivating a sense of belonging to a university and the chosen program can be challenging, especially for distance learners [30]. In the qualitative study by Brown et al. [5], half of the 20 respondents reported struggles during the initial few weeks of starting distance learning. The interviewees reported that they encountered challenges in balancing their studies with other aspects of their lives. This led to recurring doubts about their decision to pursue distance learning and, in some cases, dropping out of the program.

In general, there is limited research on the transition to distance education, resulting in a lack of understanding about the experiences, expectations, needs, and other factors that distance learners encounter during this transition period. Furthermore, there is currently no systematic overview of the limited existing empirical evidence in this field. Therefore, the proposed literature review aims to systematically examine existing published sources and provide insights into the process of the transition phase in the context of distance education. This is highly significant given the growing importance of distance learning in higher education environments, especially with the impact of the Covid-19 pandemic, as well as the crucial role of the transition to higher education in achieving successful academic outcomes.

A search for existing literature reviews on the topic of transition into distance education was conducted through the University’s Library and Google Scholar (search date: 09/13/2023). The search did not reveal any reviews previously conducted on this specific topic. The objective of the scoping review is thus to identify and analyze available literature on the study entry phase of distance learners and present the findings in a structured manner. It seeks to address the research question "What is known about transition into distance education in higher education?" within the scope of the planned review. Furthermore, relevant concepts and terminologies of the research area will be identified and classified, theoretical and empirical insights will be obtained, and the content-related boundaries will be defined. As a result of the findings, we will derive implications for practice that are intended to provide guidance for lecturers and students to promote successful digital learning. The present manuscript is the a-priori developed study protocol, which outlines the planned methodology for conducting the Scoping Review in a detailed and comprehensive manner. The drafting of a study protocol prior to performing the review is mandatory, but publication is voluntary for Scoping Reviews. The aim of publication is to increase transparency and traceability, and to minimize reporting bias [35].

## Methods

Scoping reviews are primarily utilized to answer broad and exploratory research questions, as this method includes all sources irrespective of their quality, unlike a systematic review. Thus, scoping reviews are especially beneficial for exploring new research fields, clarifying essential concepts, or identifying research gaps [31, 32]. As we aim to provide an overview of existing literature on the under systemized topic of study entry in distance education, we decided to undertake a scoping review. To ensure a transparent and comprehensible approach, the research question, aims, and methods will be proactively documented using the protocol presented here. To do so, we will follow the PRISMA-Scr (Preferred Reporting Items for Systematic Reviews and Meta-Analyses-Extension for Scoping Reviews) guidelines [33], as seen in the supporting information (S1). We will conduct the review in line with the recommendations suggested by Peters et al. [34] from the JBI research organization, as presented in Table 1. The steps to be undertaken are provided in detail below, following this approach [34, 35].

**Table 1:** Steps to conduct a scoping review according to Peters et al. 2020.

Step 1	Defining and aligning the objective/s and question/s
Step 2	Developing and aligning the inclusion criteria with the objective/s and question/s
Step 3	Describing the planned approach to evidence searching, selection, data extraction, and presentation of the evidence
Step 4	Searching the evidence
Step 5	Selecting the evidence
Step 6	Extracting the evidence
Step 7	Analysis of the evidence
Step 8	Presentation of the results
Step 9	Summarizing the evidence in relation to the purpose of the review, making conclusions and noting any implications of the findings

### Definition of the inclusion and exclusion criteria

A crucial aspect of a review is the creation of comprehensive and well-considered inclusion and exclusion criteria. Based on these, we will decide whether we will disregard sources found or consider them as part of the review process. It is recommended to formulate the research question and develop the inclusion and exclusion criteria using the PCC (population or participants/concept/context) framework when conducting a scoping review, as this captures the most significant aspects [35]. In our review we will use the following criteria:

Population/participants: The scoping review will encompass students who are enrolled in an undergraduate distance learning program. Students of on-campus courses or participants in advanced training courses not equivalent to a bachelor’s degree will be excluded. However, sources featuring pertinent statements about students transitioning to distance learning, from significant stakeholders like academic advisors or lecturers, will also be included.

Concept: The concept of the proposed review will focus on the transition or entry phase into a distance learning program. In the context of this review, the so-called study entry period will include the first two semesters of an undergraduate bachelor’s program [36]. Additionally, we will include studies focusing on the immediate period preceding the start of study, for instance, the interim period between enrollment and the first courses. Sources addressing these phases will be incorporated, while evidence pertaining to other academic periods will be excluded.

Context: The context, or setting, will be distance education. This refers to a course of study leading to a qualification that is largely completed remotely, not on a campus, unlike a traditional course of study. The transmission of the learning material, communication with the university and many other aspects are primarily carried out digitally. This mode of study offers location flexibility and self-directed time management, primarily organized around self-study. It can be blended with face-to-face meetings or phases. The proportion of in-person phases can vary. However, we will not include sources that pertain to on-campus classes or advanced training courses (whether in presence or digital) that do not lead to an academic degree.

We will consider all sorts of sources and study types that address the above inclusion and exclusion criteria. This includes scientific articles, books, grey literature, conference papers, and dissertations. Thus, no restriction is made about study types or quality to obtain the largest possible overview of the existing literature in both the national and international space. Although low quality will not lead to the exclusion of sources, we intend to critically evaluate and comment on the studies’ quality. Further, articles that are available as full text in German or English will be considered. This can be explained by the language skills of the researchers. Nowadays, most distance learning programs are conducted digitally. While there were initial online learning offerings before the 1990s, substantial progress was observed, particularly from the mid-1990s onwards (e.g., the introduction of Virtual-U field trials, a large web-based learning environment, in 1996) [37]. Though we do not want to restrict ourselves solely to digital distance learning courses, we see a temporal break here. We believe it makes sense, given this historical development, to consider sources from 1996 onwards. We will exclude sources published before 1996.

### Determination of the search strategy and study selection

Preparing a scoping review depends on the use of a search strategy built around pertinent keywords and search terms [35]. These have already been identified and are shown in Table 2.

**Table 2:** Keywords.

	P (Population/participants)	C (Concept)	C (Context)
**German**	Fernstudierende, Fernstudenten, Studierende, Studenten	Studieneingang, Studienbeginn, Studieneintritt, Studienanfang, Studieneingangsphase, Einführungsphase, Eingang, Beginn, Eintritt, Übergang, erstes Semester, erstes Jahr	Fernstudium, Fernstudien, Online-Studium, Fernunterricht virtuell, digital,
**English**	Students, undergraduates	entrance, entry, start, transition, introductory phase, launch phase, initial phase, adaption, adaptation, adjustment, first semester, first year	distance education, distance learning, correspondence course, correspondence degree course, online, virtual, remote, distance, digital, e-learning

The list was compiled following an initial search of the University’s library and Google Scholar. Essential keywords and phrases were gleaned from the articles discovered. Next, we conducted a search for terms used synonymously to yield a robust list of relevant keywords. We performed this process in both German and English. The identified keywords are used to formulate search strings for the databases to be screened. This is achieved by applying common Boolean operators and, if necessary, using wildcards. A total of six databases will be searched for suitable articles: ERIC (Education Resources Information Center), Pubmed, Google Scholar, PsycINFO, Scopus, and BASE (Bielefeld Academic Search Engine). The chosen databases are comprehensive and widely used, covering relevant subdomains such as educational science, psychology, health sciences, or serving as resources for general literature searches. We assume that we have chosen the most pertinent databases and believe that they will provide us with most of the sources that are of interest to us. Each database will be examined independently by one author utilizing the specific search strings. As an example, we created the following string for the Pubmed database:

*(students[Title] OR undergraduates[Title]) AND (entrance[Title] OR entry[Title] OR start[Title] OR transition[Title] OR "introductory phase"[Title] OR "launch phase"[Title] OR "initial phase"[Title] OR adaption[Title] OR adaptation[Title] OR adjustment[Title] OR "first semester"[Title] OR "first year"[Title]) AND ("distance education"[Title] OR "distance learning"[Title] OR "correspondence course"[Title] OR "correspondence degree course"[Title] OR online[Title] OR virtual[Title] OR remote[Title] OR distance[Title] OR digital[Title] OR e-learning[Title])*

A search of Pubmed using the created search string resulted in 122 potential sources, given the search was limited to the title as well as the timeframe from the year 1996. A title-related search seems reasonable since the term "online" would otherwise yield all sources that used an online questionnaire for data collection. Without this limitation, the same search string yields 9954 potential sources, most of which are outside the scope of our research interest.

The studies identified through this process will first be screened by two authors for relevance and alignment with inclusion criteria based on the title and abstract. They will be excluded or considered for further review on a case-by-case basis. If disagreements arise, a third reviewer will be consulted. Next, we will check all remaining titles for duplicates, which will be removed if necessary. In a next step, the reference lists of the articles found will be searched for additional sources, which again will be revised by two reviewers based on the inclusion and exclusion criteria. Finally, each paper that is left will be reviewed independently by two authors for relevance and fulfillment of the inclusion criteria based on the full text. In case of disagreement between the reviewers, a third person will be consulted. The implementation of these steps will result in a specific number of articles to be included. The search and selection of studies is an iterative process, which means that the search strategy can be continuously updated throughout the process. We will use reference management software, such as Zotero, to create a database of all relevant articles.

### Data extraction

Following the search strategy we will extract relevant content using a table that captures essential information related to the research question and other information, such as the year, sample, and study design. An initial draft of this form is presented in Table 3. Further refinement and modifications may be made during the scoping review process. We will perform the data extraction using Microsoft Excel.

**Table 3:** Extraction form.

Source in accordance with APA 7th edition	Aim	Context of distance learning	Population and sample size	Methodology or methods	Key findings	Distance learning solely due to the Covid pandemic? (Emergency remote teaching)

### Analysis of the data

The relevant results will be analyzed according to Kuckartz’s [38] qualitative content analysis using MAXQDA software. This rule-based procedure seems especially suitable and practical due to the collaborative efforts of multiple authors in the data analysis. Furthermore, the question-based nature of this method, which enables data to be reduced via a category-based system [39], appears beneficial for our planned approach. Ultimately, an inductive category system will be developed to depict the experiences of distance learners in the beginning of their studies.

### Presentation of results

**Fig 1:**
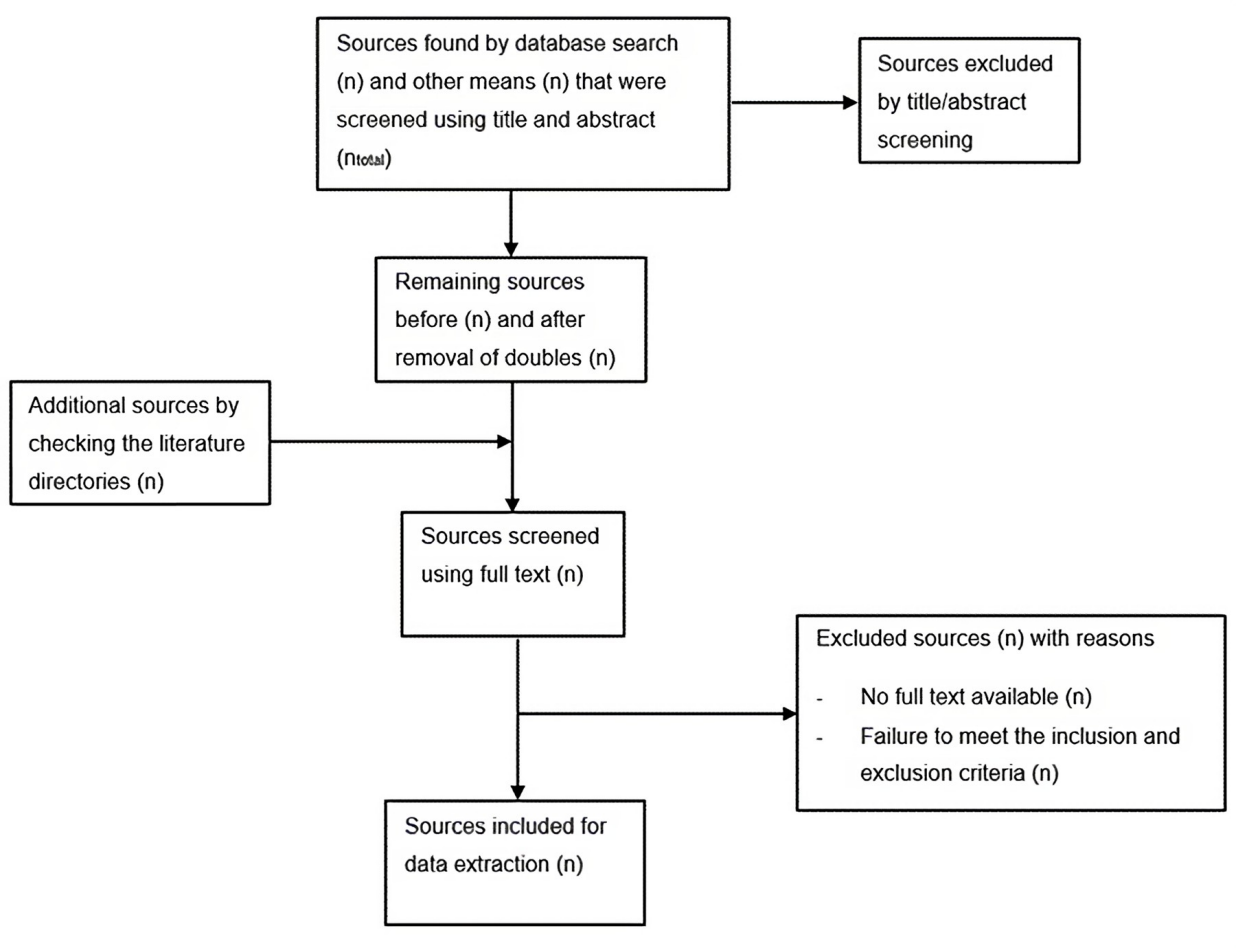
Search flowchart following PRISMA guidelines.

We will first describe the literature search and study selection process both narratively and visually, using a flowchart in accordance with the PRISMA-Scr recommendations [33]. Refer to Fig 1 for further details.

Following that, the presentation of the results will include a descriptive summary that links the findings to the research question and the objectives of the review. The outcomes, including key concepts and categories, may also be visualized in table or map format. We will ensure the presentation of results conforms to PRISMA-Scr guidelines [33].

## Limitations

The planned investigation is constrained by limitations. For example, due to personnel and time resources, we must limit the number of databases we search to a certain number. This means that any content not indexed in these databases will not be processed. Due to our language skills, we can also only search for articles that are available in full text in English or German. Studies published in other languages will therefore not be considered. Furthermore, the fact that we will not assess the quality of the sources may lead to the inclusion of studies that have certain deficiencies in their design.

## Discussion

The demand for and supply of distance learning programs are consistently increasing. Concurrently, it is recognized that the transition to higher education significantly influences subsequent study success or non-success [20]. Yet, there is scarce knowledge about this critical phase in particular in relation to distance learning. Brown et al. [5] provide insight into the unique experiences of students enrolling in distance education for the first time. While some students adapted successfully to the new demands, half of the distance learners reported significant challenges during the initial weeks of their studies. This was largely attributed to the difficulty of incorporating the new demands of distance learning into their existing lives. Nonetheless, it is the flexibility of distance education that offers students with family responsibilities or who are in employment, for instance, the ability to pursue a higher education degree [6].

Undertaking distance education holds new challenges. For example, distance learners must possess a high capacity for self-directed learning along with a robust sense of personal responsibility. Factors such as delayed feedback, limited access to resources and technology, physical isolation, and sometimes considerable tuition fees can adversely affect the transition to distance education [6]. While distance education providers cannot impact all the factors that are crucial to a successful entry phase and ultimately to study success, Brown et al. [5] emphasize the importance for these institutions to take into account students’ needs—for example, when designing courses or devising targeted interventions and support services. For instance, Aristeidou’s study [40] indicates that students primarily find a fixed course structure, university-provided support, regular communication with lecturers, a gradual introduction to the course, and varied tutorials helpful during the transition to distance learning. Students particularly expressed a need for enhanced communication and interaction opportunities to foster social relationships with their peers.

However, to date, there appears to be a lack of systematic gathering, assessment, and presentation of the current research state regarding the transition to distance education, including the experiences, challenges, adaptation strategies, and living situations of distance learners. This gap is now to be closed by the planned scoping review with the aim of defining the content-related boundaries of the topic "transition into distance education" as well as identifying and classifying the relevant concepts and terms of the research field. We anticipate that the review results will offer valuable, in-depth insights into the studied domain. The insights gathered from the scoping review will offer theoretical clarity, compile the existing theoretical and empirical understanding in the field, and provide practical recommendations for distance education providers and lecturers, which they might want to take into account when planning distance education programs in the future.

## Supporting information

S1 File PRISMA-P (Preferred reporting items for systematic review and meta-analysis protocols) checklist. (PDF)
